# Diagnostic indicators and lifestyle interventions of metabolic-associated fatty liver disease

**DOI:** 10.3389/fnut.2024.1424246

**Published:** 2024-06-14

**Authors:** Tianzhu Chen, Xiang Qin, Jianping Jiang, Beihui He

**Affiliations:** ^1^The First Affiliated Hospital of Zhejiang Chinese Medical University (Zhejiang Provincial Hospital of Chinese Medicine), Hangzhou, China; ^2^Hangzhou Lin’an Traditional Chinese Medicine Hospital, Affiliated Hospital, Hangzhou City University, Hangzhou, China; ^3^School of Life Sciences, Zhejiang Chinese Medical University, Hangzhou, China

**Keywords:** metabolic dysfunction-associated fatty liver disease, biomarker, lifestyle interventions, diet, activity

## Abstract

MAFLD has become a major global health problem and is the leading cause of liver disease worldwide. The disease progresses from a simple fatty liver to gradual fibrosis, which progresses to cirrhosis and even hepatocellular liver cancer. However, the methods currently used for diagnosis are invasive and do not facilitate clinical assessment of the condition. As a result, research on markers for the diagnosis of MAFLD is increasing. In addition, there are no clinical medications for the treatment of MAFLD, and lifestyle interventions remain effective in the prevention and treatment of MAFLD. In this review, we attempt to make a summary of the emerging diagnostic indicators and effective lifestyle interventions for MAFLD and to provide new insights into the diagnosis and treatment of MAFLD.

## 1 Introduction

In 2020, the hepatology community introduced the novel term “metabolic dysfunction-associated fatty liver disease” (MAFLD) to replace “non-alcoholic fatty liver disease” (NAFLD) (1). Recently, this term was again replaced by “metabolic dysfunction-associated steatotic liver disease” (MASLD), along with a revised definition of metabolic dysfunction (2). MAFLD is a common chronic liver disease worldwide. The high prevalence of MAFLD has been noted in several reports (3–5). Overall prevalence is statistically increasing steadily and is projected to reach 55.7% by 2040 (6). MAFLD is a type of liver injury characterized by increased fatty deposits in hepatocytes with varying histological severity and the potential to progress to fibrosis, cirrhosis, and hepatocellular liver cancer. Fibrosis stage was the only independent predictor of long-term prognosis in MAFLD. Liver biopsy remains the gold standard for the diagnosis of liver fibrosis, but because it is an invasive test that is difficult to use frequently in the clinic, it has become important to find a noninvasive index for the effective assessment of MAFLD. In addition, there are no approved medications for the treatment of MAFLD, and lifestyle interventions continue to be the most recognized method of preventing and treating MAFLD.

The purpose of this review is to summarize current diagnostic indicators and effective lifestyle intervention methods for MAFLD.

## 2 Diagnostic indicators

### 2.1 Liver fat content

Magnetic Resonance Proton Density Fat Fraction (MRI-PDFF) has the ability to quantify hepatic fat over the entire dynamic range. It is a more accurate quantitative imaging biomarker that can be used to evaluate liver fat content. In a meta-analysis (*n* = 6 studies in 635 patients with biopsy-proven NAFLD), the summary AUROC values of MRI-PDFF for detecting steatosis S0 and S1–S3 (≥5%), S0–S1 and S2–S3 (≥33%), S0–S2 and S3 (≥66%) were 0.98, 0.91, and 0.90, respectively. Pooled sensitivity and specificity were 93 and 94%, 74 and 90%, and 74 and 87%, respectively (7). However, due to its high cost, it is not recommended for routine clinical use.

Controlled attenuation parameter (CAP) is the algorithm available on the FibroScan system (Echosens, Paris, France) for quantification of the liver fat content. This is a convenient and readily available modality that has been widely used to assess liver fat content. However, several covariates such as NAFLD, diabetes mellitus, and BMI affect CAP values. Currently, guidelines consider values above 275 dB/m to have high sensitivity and PPV (>90%) in NAFLD, although there is no uniform cut-off value for CAP (8). One study summarized that in healthy adolescent boys/girls, the upper limit of normal for CAP is 258.9/243.1 dB/m (12–13.9 years old), 251.9/266.3 dB/m (14–15.9 years old), 247.0/265.2 dB/m (16–17.9 years old), and 249.3/246.0 dB/m (18–19.9 years old) (9). Recent studies have analyzed that in children with NAFLD, using MRI-PDFF grading as a criterion for steatosis, the optimal cutoffs for CAP in the subgroups of S0 vs. S1–S3 (≥6.4%), S0–S1 vs. S2–S3 (≥17.4%), and S0–S2 vs. S3 (≥22.1%) were 265 dB/m, 299 dB/m, and 303 dB/m, respectively (10).

Recently, a study constructed a new CAP-based scoring scale, CBST.CBST = −14.27962 + 0.05431 × CAP −0.14266 × BMI + 0.01715 × AST. Confirmed that CBST scores are more accurate than CAP itself. The optimal CBST cutoff values for MRI-PDFF diagnosis of ≥20%, ≥10%, and ≥5% were −0.5345 (sensitivity = 72.1%, specificity = 70.8%, PPV = 53.7%, NPV = 84.4%), −1.7404 (sensitivity = 88.7%, specificity = 76.0%, PPV = 91.2%, NPV = 70.4%) and −1.9959 (sensitivity = 86.4%, specificity = 92.9%, PPV = 99.4%, NPV = 35.1%) (11). This would be a practical diagnostic basis if more studies were available to verify its accuracy.

### 2.2 Fibrosis score

#### 2.2.1 Fibrosis-4 score, the aspartate aminotransferase-to-platelet ratio index

The FIB-4 and APRI is a score that uses routine laboratory parameters to assess liver fibrosis, and the formula is FIB-4 score = age(year) × AST(U/L)/[PLT(109/L) × ALT1/2(U/L)] APRI = [AST(U/L)/(AST(upper limit of normalcy)(U/L)]/PLT(109/L) × 100). It has been shown that it can be used to assess the degree of hepatic fibrosis in chronic hepatitis B patients. The sensitivity and specificity of the APRI score > 0.342 to differentiate between patients with “no and mild fibrosis” and those with “severe fibrosis” were 63 and 64%. Respectively, and the sensitivity of the FIB-4 score > 0.70 to differentiate between patients with “no and mild fibrosis” and those with “severe fibrosis” was 71%, with a sensitivity of 71% (12). It has been noted that despite the high diagnostic accuracy of FIB-4 and APRI for advanced fibrosis in patients with NAFLD, their ability to diagnose hepatic fibrosis in its early stages remains controversial (13). Furthermore, it was shown that the APRI (AUROC range 0.73–0.80) and FIB-4 (AUROC range 0.66–0.82) outperformed the NAFLD fibrosis score (NFS) (AUROC range 0.63–0.75) in predicting liver fibrosis in MAFLD (14). In a meta-analysis of more than 40,000 participants, it was noted that when used to predict the severity of liver fibrosis in patients with MASLD, FIB-4 had good predictive diagnostic accuracy for any fibrosis, and FIB-4 had good diagnostic accuracy for cirrhosis, and could be used to assess the prognosis of MASLD (15).

#### 2.2.2 MAFLD screening score, fatty liver index, hepatic steatosis index

Detection of hepatic steatosis HS is a mandatory criterion for the diagnosis of MAFLD, and the development of a non-invasive means of rapid and standardized detection of HS has become very important. Han et al. retrospectively evaluated the performance of FLI and HSI with respect to the prediction of MAFLD diagnosed by computed tomography (CT), and FLI (AUROC, 0.793) and HSI (HSI 0.784) were feasible in the prediction of MAFLD (16). In a large cross-sectional survey in China including 135,436 patients, the FLI AUROC used to predict MAFLD for male and female patients was 0.870 and 0.923 (17). A Mexican population-based study developed a score, MAFLD-S, for the prediction of MAFLD with an AUC of 0.852, 95% CI = 0.828–0.877, an optimal cutoff of 0.548, a sensitivity of 78.8%, and a specificity of 82.8% (18). In a cross-sectional study, assessment of MAFLD-S, FLI, and HSI is a valid tool to accurately predict MAFLD in patients with inflammatory bowel disease IBD (19). Valuable tool for FLI prediction of MAFLD noted in Japanese population-based survey (20). In a cross-sectional study based on adults in Xinjiang, FLI, and HSI had good screening for MAFLD in both men and women, with FLI having the best screening ability (21). FLI and HSI have excellent discriminatory ability in predicting MAFLD in the general population, both in the general population and in individuals at metabolic risk, FLI and HIS will be effective tools for predicting and screening for MAFLD (22).

#### 2.2.3 TyG-body to mass index, TyG-waist circumference

A cross-sectional study of people aged 25–75 years showed that TyG-WC, TyG-BMI were the best predictors of MAFLD, independent of age, sex, obesity or diabetes status (23). Some studies have shown that TyG-BMI has good diagnostic efficacy in identifying people at risk for MAFLD in western China. In contrast, TyG-WC had the best diagnostic performance for identifying MAFLD risk in the US population. These findings suggest the need to select the most appropriate predictive model based on regional and racial differences (24). A Korean population-based study suggests that TyG-WC, TyG-BMI are positively correlated with MAFLD risk and can be used clinically to rapidly identify patients at risk of MAFLD (25).

### 2.3 Serum biomarker of MAFLD

#### 2.3.1 Chitinase-3-like protein 1

Chitinase-3-like protein 1 (CHI3L1, YKL-40 protein) is a glycoprotein that is abundantly expressed in liver tissues and is mainly involved in inflammation and tissue remodeling (26). It has been shown that CHI3L1 is significantly elevated in chronic liver diseases such as HBV-Related Liver Diseases, HCV-Related Liver Diseases, and hepatocellular carcinoma, and is associated with the degree of fibrosis (27–31). Studies have shown that CHI3L1 is an indicator used to assess fibrosis, and Xiaoting Huang el confirmed that serum CHI3L1 is a viable indicator for measuring liver fibrosis. The AUC for serum CHI3L1 was 0.812, *p* < 0.000. The Youden index was highest at a serum CHI3L1 level of 83.36 ng/mL, with a sensitivity of 88.2% and a specificity of 66.4% (32). Yanqiang Liao el demonstrated that CHI3L1 detected by chemiluminescent immunoassay (CLIA) has good diagnostic value for HBV-associated hepatocellular carcinoma (27). It has been shown that CHI3L1 is a good biomarker in MAFLD and assesses the risk of severe liver fibrosis. The AUC for CHI3L1 in the diagnosis of significant liver fibrosis was 0.716 (95% CI, 0.596, 0.836), with a corresponding optimal cutoff value of 125.315 ng/mL (33). High levels of serum CHI3L1 in patients with type 2 diabetes may indicate liver fibrosis in patients with MAFLD, CHI3L1 showed the area under the ROC curve (AUC) for detection of significant fibrosis (0.749, 95% CI, 0.668–0.829, *p* < 0.001), and the value of 94.89 ng/mL (sensitivity:54.4%, specificity:87.6%, *p* < 0.001) was the best cutoff point to predict significant liver fibrosis in T2DM-MAFLD patients (34).

#### 2.3.2 Leukocyte cell-derived chemotaxin-2

LECT2 is a chemokine (35), it is mainly produced by the liver. It has been shown that LECT2 attenuates fatty changes and insulin resistance in the liver (36). Whereas insulin resistance is a key factor in the development of MAFLD, LECT2 may be involved in the development of MAFLD. It has been shown that LECT2 levels were 31.2 (20.9, 41.5) ng/mL in the group of people with NAFLD compared to those without NAFLD, which was significantly higher than in the group without NAFLD (37). It is proposed that LECT2 is highly expressed in both hepatic tissue and serum in biliary atresia (BA), suggesting that LECT2 can be used as a biomarker for BA (38). LECT2 levels are increased in patients with metabolic syndrome (MetS) and correlate with severity, suggesting that LECT2 can be used as a biomarker for MetS (39). Currently, there are experiments showing an increase in serum LECT2 concentration in children with NAFLD, which showed a diagnosis of NAFLD at a concentration of 3.76 ng/mL with a sensitivity of 90.5% and a specificity of 54.8%, suggesting that LECT2 can be used as a diagnostic biomarker for MAFLD in children (40), which has not been studied in adults with MAFLD.

#### 2.3.3 Cathepsin D

Cathepsin D (CTSD) is a lysosomal enzyme involved in inflammatory responses and lipid metabolism, and previous data have shown that hepatic inflammation is associated with hepatic CTSD activity and expression (41, 42). In addition, plasma CTSD concentrations were associated with different stages of NAFLD, Significantly elevated in metabolic dysfunction-associated steatohepatitis (NASH) (43). A recent study analyzed RNA sequencing of MASLD and found higher expression of CTSD in severe MASLD compared to controls and mild disease, and validated the diagnostic role of serum CTSD in MASLD, showing that CTSD contributes to the accuracy of FIB-4 in diagnosing MASLD. The AUC of serum CTSD in predicting NASH fibrosis was 0.731 with a cutoff value of 10624.5 pg./mL (specificity 47.1%, sensitivity 93.3%) (44).

#### 2.3.4 Plasminogen activator inhibitor-1

Plasminogen activator inhibitor-1 (PAI-1) is the most important inhibitors of the plasminogen/plasmin system (45). PAI-1 plays an important role in hepatic fibrogenesis (46). It has been shown that elevated plasma levels of PAI-1 correlate with the degree of hepatic steatosis (47). The study found that the PAI-1 gene was significantly down-regulated in NAFLD by bioinformatic analysis of the GEO database. Although bias may be induced by the small sample size, it suggests that PAI-1 has the potential to serve as a diagnostic marker for NAFLD (48). Recent studies have found that as liver pathology progresses toward nonalcoholic steatohepatitis (NASH), there is a corresponding increase in the level of mRNA expression of Serpine1, as well as the protein level of PAI-1. Use of Serum PAI-1 Levels as a noninvasive biomarker to identify NASH-associated fibrosis (49). However, no experiments have been performed to assess its diagnostic value in MAFLD.

## 3 Lifestyle interventions of MAFLD

### 3.1 Healthy diet

#### 3.1.1 Daily dietary patterns

**Figure 1:**
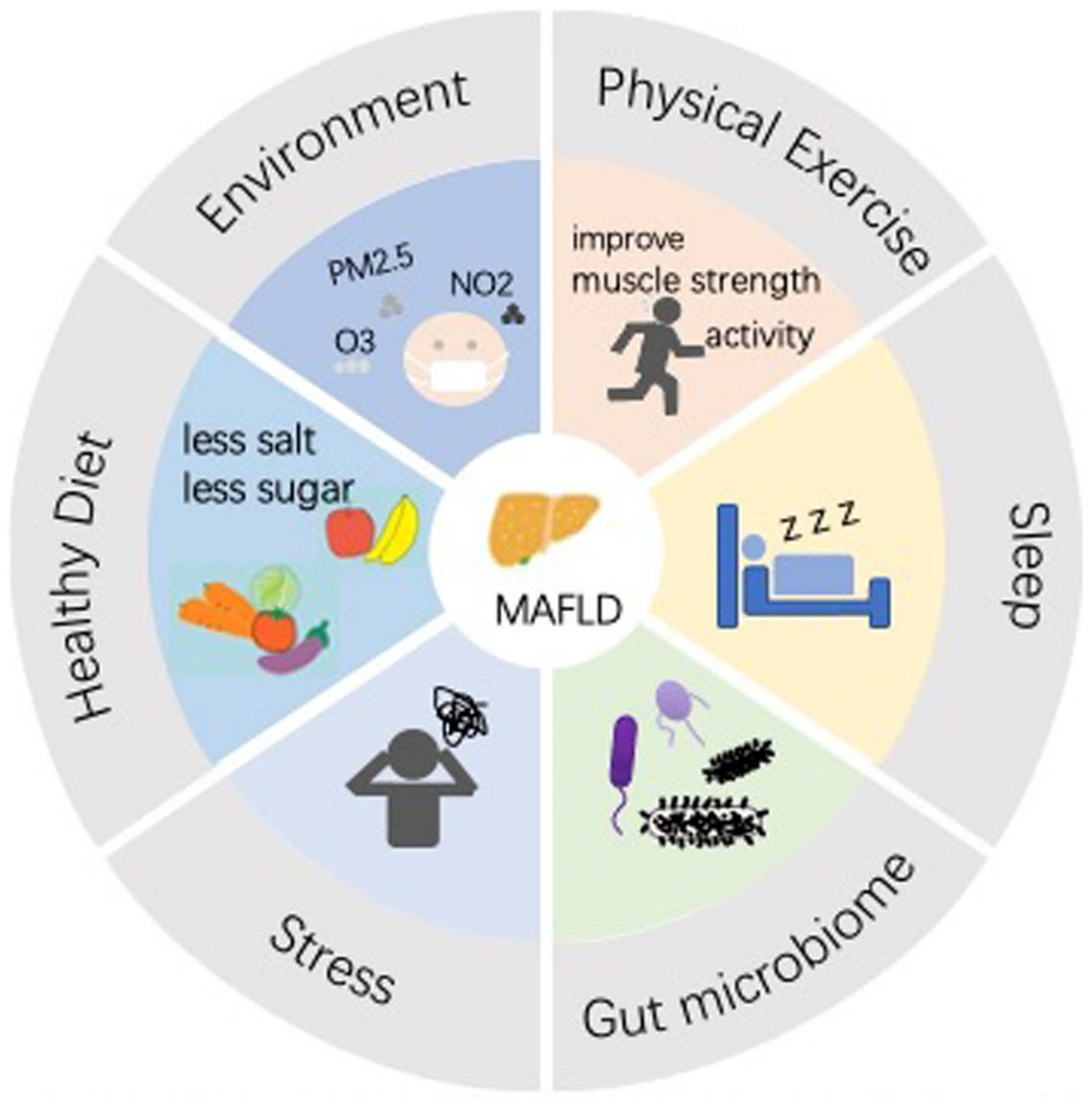
Lifestyle interventions are now a widely recognized method of prevention and treatment for MAFLD. An effective lifestyle includes a proper diet (less salt, less sugar, good quality carbohydrates, unsaturated fatty acids, vitamin D, etc.), and good habits (exercise, adequate and good quality sleep, good mood, improved gut microbiology, reduced air pollution, etc.).

Moderate fiber intake is associated with lower odds of MAFLD compared to low fiber intake (50). Healthy low-carbohydrate and low-fat diets are protective against MAFLD, while unhealthy low-fat diets have deleterious effects on MAFLD (51). A study based on a Korean population showed that a dairy-rich dietary pattern was associated with a lower risk of MASLD, The cumulative incidence of MASLD was also significantly lower when adhering to a dietary pattern rich in dairy products (52). βHB, one of the ketone, has been reported to have inhibitory effects on adipocyte lipolysis, liver fat accumulation and inflammatory responses, suggesting a possible protective effect against MAFLD (53). The ketogenic diet (KD) is a diet that is very low in carbohydrate intake, and KD can be beneficial in treating diseases such as MAFLD and NASH. Sugar deficiency markedly reduces the effect on insulin resistance, and despite the benefits of KD, precautions need to be taken regarding the nature of dietary fats, and saturated fatty acids can be replaced with polyunsaturated fatty acids (54). One study tried to assess the effects of 8 weeks of a very low-calorie ketogenic diet (VLCKD) on MASLD, and found that it significantly reduced liver stiffness, and substantially reduced WC, fat mass, systolic and diastolic blood pressure, and BMI, and effectively reduced fasting glucose, insulin, insulin resistance (measured by HOMAIR), triglycerides, total cholesterol, LDL cholesterol, HDL cholesterol, and GT. This suggests that VLCKD improves insulin sensitivity and results in elevated levels of vitamin D. VLCKD treatment also reduces low-grade inflammation (55) (Figure 1).

The Mediterranean diet is currently the more recommended diet for the prevention of MAFLD (56). The Mediterranean diet includes plenty of vegetables, fruits, legumes, nuts and olive oil, as well as small to moderate consumption of red meat and wine, which has anti-inflammatory, antioxidant and antifibrotic components. Adherence to the Mediterranean diet, especially higher fruit intake, is associated with lower severity of MAFLD (57). Additional studies have found that the Mediterranean diet DM and the Mexican regional diet RMD are equally effective in improving MASLD symptoms, particularly steatosis, highlighting the feasibility of using regionally adapted diets to treat fatty liver. DM implementation is not feasible in all countries. Therefore, adapting the diet to the ingredients available in that country is a better option, as balanced implementation can improve the health of patients with fatty liver disease (58).

In China, three regional dietary patterns have been evaluated for their association with MAFLD: the Sichuan Basin pattern, characterized by high intakes of fish/seafood, poultry, fresh fruits and vegetables; the Yunnan-Guizhou Plateau pattern, characterized by high intakes of animal oils and salt; and the Qinghai-Tibet Plateau pattern, characterized by high intakes of coarse grains, wheat products, tubers and tea. The results showed that both the Yunnan-Guizhou Plateau dietary pattern and the Tibetan Plateau dietary pattern were positively associated with MAFLD. Studies point to an undesirable role of salt, animal oils and high carbohydrates in the progression of MAFLD, with animal oils rich in saturated fatty acids (SFA) being positively associated with MAFLD (59).

In addition, a vegetarian diet is now considered a healthy diet, and a vegetarian diet means replacing meat and fish with soy and refined carbohydrates with whole grains. Studies have shown that vegetarian diets may be associated with less pronounced liver fibrosis and that vegetarians have lower NAFLD fibrosis scores, which could mean lower cardiovascular-related mortality in the future (60). However, there has been no extensive research on its relationship with MAFLD progression, incidence or prevalence (61).

Therefore, a dietary pattern with less salt, less sugar and more vegetables and fruits has a favorable effect on the prevention of MAFLD.

#### 3.1.2 Dietary supplement

In addition to daily dietary changes that are beneficial in preventing MAFLD, increasing or decreasing the intake of some dietary supplements may also be beneficial in reducing the incidence of MAFLD. Studies have shown that high dietary selenium intake increases the risk of MAFLD by modulating insulin biosynthesis and secretion dysregulation as well as stimulating glucagon secretion, insulin resistance and dyslipidemia (62). Reducing the ratio of Se and vitamin E, or not supplementing them at all, may reduce the prevalence of MAFLD (63). Patients with MAFLD consume more vitamin E in their daily diet than healthy controls, so increased vitamin E may be positively associated with MAFLD (64). Thus suggesting that to prevent MAFLD, we need to reduce se and vitamin E intake.

Patients with MAFLD consume more polyunsaturated fatty acids PUFA and iron in their daily diets than healthy controls Increased intake of these substances may be positively associated with MAFLD (64). According to an analysis of NHANES 2017–2020 data, high copper intake and moderate iron intake are associated with low odds of MAFLD (65). Therefore, we believe that there is also a relationship between copper and iron intake, and the occurrence of MAFLD. A significant association between vitamin D insufficiency and increased incidence of MAFLD suggests the potential of vitamin D as an anti-adipogenic and anti-fibrotic agent (66). A randomized controlled trial of vitamin D supplementation on serum levels of VDR, fibrotic factors and fibrotic microRNA (MiR) levels in patients with MASLD revealed for the first time significant reductions in some hepatic fibrotic factors, hepatic aminotransferases, and the corresponding changes in some fibrotic-associated MiR and some metabolic factors (67). suggests that vitamin D supplementation is beneficial in reducing the likelihood of MASLD fibrosis.

MAFLD is primarily caused by fat accumulation, and oral glutamine supplementation leads to insulin resistance in fat cells, which reduces fat (68). Glutamine was shown to contribute to attenuating the severity of hepatic lipid injury in mice exposed to high-fat diet (HFD) induced MAFLD by ameliorating changes in serum lipids, hepatic lipid metabolism, and oxidative stress (69).

Silymarin is a flavonoid compound derived from milk thistle seed that has been used for many years, as a Chinese herbal treatment for liver diseases (70). Silymarin can not only promote the production of glutathione to enhance the ability to resist oxidative stress (71), but also can inhibit the expression of NF- κ B, reduce TNF- α, IFN- γ, IL-2, and IL-4, and reduce the inflammatory response (72). In addition, silymarin can improved glucose tolerance and insulin tolerance in NAFLD patients, and the colonizing of altered microbiota from silymarin and polyherbal extract treated mice directly ameliorated NAFLD (73).

Insulin resistance is a common pathological feature in MAFLD patients, and artichoke has been found to have positive amelioration on insulin resistance. Previous studies have shown that water extract from artichoke ameliorates high-fat diet-induced non-alcoholic fatty liver disease in rats (74). Deng’s study (75) demonstrated that artichoke water extract (AWE) reduced the expression of acid enol phosphoenolpyruvate carboxykinase (PEPCK) and glucose-6-phosphatase (G6Pase), and inhibited insulin resistance to improve glucose metabolism. Meanwhile, AWE also inhibited the endoplasmic reticulum stress to protect HepG2 cells. This is thought to be the resulting from the regulation of IRS 1/PI3K/AKT/FoxO 1 and GSK-3 β signaling.

Bergamot is a citrus fruit with extensive biological activity which has anti-proliferative, pro-apoptotic, anti-inflammatory and anti-oxidation effects to make positive influence on MAFLD (76). Bergamot extract can reduce blood glucose levels in type 2 diabetic mice, prevent hyperglycemia caused by leptin receptor disruption, and regulate glucose homeostasis by enhancing insulin sensitivity. In this study, Liu et al. (77) also observed reduced TC, TG, and LDL-C levels, which effectively controlled blood lipids and protected the liver. In NAFLD, bergamot extracts can decrease the hepatic steatosis (78).

*P. tenuifolia* seed oil (PWSO) therapeutic diet (17% lard and 14% PWSO diet) inactivates SREBP1 and SREBP2, which are involved in lipogenesis, to attenuate hepatic lipid accumulation and reduce inflammatory responses induced through the NF-κB signaling pathway. Studies have shown that PWSO can be a relatively effective dietary supplement to inhibit the onset and progression of MAFLD (79).

We believe that moderate dietary supplements are beneficial for the prevention of MAFLD (Table 1).

**Table 1:** Effect of dietary supplements in MAFLD.

Author (reference)	Supplement	Experimental model	Concentrations of supplement	Findings
Niu et al. (64)	Polyunsaturated fatty acids (PUFAs); vitamin E; Iron	MAFLD patient	The percentages of individuals consuming PUFAs for >11% of their total energy intake, >14 mg/day of vitamin E, and >12 mg of iron	Risk of MAFLD↑
Guo and Yu (63)	γ-tocopherol; α-tocopherol; Se	MAFLD patient	serum concentrations of γ-tocopherol (1.43 ± 0.52 μmol/L), α-tocopherol (3.38 ± 0.31 μmol/L) and Se (0.89 ± 0.13 μmol/L)	Risk of MAFLD↑
Hou et al. (65)	Copper; Iron	MAFLD patient	Copper intake > 0.53 mg/1,000 kcal, Iron intake 5.19–7.57 mg/1,000 kcal	Risk of MAFLD↓
Lee et al. (66)	Vitamin D	MAFLD patient	Serum vitamin D levels of 14.07 ± 3.55 ng/mL (men); Serum vitamin D levels of 12.57 ± 3.98 ng/mL (women)	Risk of MAFLD↑
Ebrahimpour-Koujan et al. (67)	Vitamin D	MASLD patient	4,000 IU/d vitamin D (12 weeks)	ALT, AST, FBS, and LDL-C levels↓ Serum 25(OH) vitamin D, VDR, and HDL-C↑ MiR-21 and MiR-122 gene expressions↓
Abboud et al. (68)	Oral glutamine	Wistar rats on a high-fat diet (HFD); Overweight (BMI ≥ 25 kg/m2) and obese (BMI ≥ 30 kg/m2) humans	Glutamine (0.4 g in 1 mL) 3 days a week for 4 weeks (rats); a total of 30 g of Gln per day, lasted for 14 days (humans); a dose of 0.4 g/kg of glutamine in humans and 2.4 g/kg in rats	Waist Circumference and Circulating LPS↓ Weight↓ Glucose incorporation in adipose tissue↓ Not increase insulin-induced Akt phosphorylation
Zhang et al. (69)	Glutamine	High-fat diet (HFD)-induced MAFLD C57BL/6 mouse model	HFD concomitant with 4% glutamine treatment for 24 weeks	Lipid catabolism↑ lipid accumulation↓ Glutamine-based treatments alone cannot reverse serum lipid dysregulation
Sozen et al. (71)	Silymarin	Adult female Wistar Albino rats	100 mg/kg/ day oral SIL for 14 days, once in 24 h	Hepato-cyte degeneration and multinuclear giant cell formation↑ Prevented DNA damage Oxidative stress in tissues ↓
Wang et al. (73)	Silymarin	High-Fat Diet-Induced NAFLD in Mice	HFD supplemented with a medium or high dose of silymarin (0.101 g, 0.202 g)	Glucose tolerance and insulin tolerance↑ Pro-inflammatory cytokines tumor necrosis factor-α (TNF-α) and interleukin-17 (IL-17) in the liver↓ altered microbiota from silymarin and polyherbal extract treated mice directly ameliorated NAFLD
Deng et al. (74)	Water extract of artichoke	Rats were fed a high-fat diet (HFD) for 8 weeks to induce NAFLD	Treated with WEA at three doses (0.4, 0.8, and 1.6 g/kg body weight, BW) for 8 weeks	Blood lipid metabolism and liver function↑ Hepatic oxidative stress↓ mRNA expression of inflammatory genes↓ Akt phosphorylation at Ser473 in the liver↑
Deng et al. (75)	Water extract of artichoke	Palmitic acid (PA)-induced IR model in HepG2 cells	AWE (containing 1.2% chlorogenic acid, 4.8% cynarin)	PA-induced lipotoxicity and IR in HepG2 cells↓
Liu et al. (77)	Soluble dietary fiber (SDF) and insoluble dietary fiber (IDF) from bergamot	Diabetic mice	Diabetic mice treated with SDF 800 mg/kg BW/day or IDF 800 mg/kg BW/day	Blood glucose levels↓ Homeostatic index of insulin resistance ↓
Maurotti et al. (78)	Bergamot and artichoke	NAFLD patient	Containing *Cynara cardunculus* extract and bergamot polyphenol fraction 300 mg/d	Hepatic steatosis↓ Peripheral vascular endothelial function in adults↑
Xin et al. (79)	*P. tenuifolia* seed oil	Rats were fed a high-fat diet (HFD, 31% lard oil diet)	PWSO treatment diet (17% lard oil and 14% PWSO diet) for 8 weeks	Hepatic levels of TC and TG↓ IL-6 levels and TNF-α level↓

#### 3.1.3 Drinks

Green tea is generally recognized as a healthful drink (80). Tea consumption (≥1 cup/day) is not associated with the prevalence of newly diagnosed NAFLD among the general Chinese adult population, and further research may be needed to examine the association between higher frequency tea consumption and MASLD, says a study based on the Tianjin population (81). A 2020 study suggested that green tea reduced liver enzyme levels in participants with non-alcoholic fatty liver disease (NAFLD), but liver enzymes were significantly increased in healthy subjects (82). This suggests to us that drinking more green tea may be effective in reducing the prevalence of MAFLD.

It has also been suggested that caffeine attenuates liver fat and stiffness in patients with diabetes and NAFLD (83). Coffee is associated with NAFLD severity in type 2 diabetics (84). For overweight/obese patients with MASLD and T2D, coffee consumption may have potential benefits (85). It has also been shown that excessive soft drink consumption is still significantly associated with MASLD (86).

Therefore, appropriately increasing the consumption of green tea and coffee and decreasing the consumption of soft drinks in daily life may help prevent MAFLD.

### 3.2 Living habits

#### 3.2.1 Physical exercise

According to data from the 2003–2006 U.S. population, longer physical activity (PA) is associated with a lower risk of cardiovascular disease-related death in patients with NAFLD (87). A study based on the 2017–2018 U.S. population shows that physical activity is strongly associated with a lower risk of obese and non-obese MAFLD (88). Active physical activity PA and adequate weekday sleep duration are both inversely associated with the risk of MASLD, and combining them can further reduce the risk of MASLD (89). Increased physical activity improves the strength of the muscles in the body. Whereas muscle strength may play a critical role in the incidence and progression of NAFLD/MAFLD, interventions to improve muscle strength in the management of NAFLD/MAFLD may be helpful (90). One study showed that adherence to an overall healthy lifestyle of non-smoking, non-alcohol use, physical activity, and healthy diet was associated with a 19% reduction in adjusted MAFLD risk (91).

It has been suggested that concentrating on physical activity 1 or 2 days per week (WW model) versus an average weekly schedule of physical activity (RA model) are both associated with lower DXA measures of fat mass (both in the abdominal region and whole body), BMI, and waist circumference. The WW model may also be applicable to the prevention of NAFLD (92).

#### 3.2.2 Sleep

Short sleep duration and poor sleep quality are significantly associated with increased risk of NAFLD, according to a Korean population-based study (93). A study of 2,172 people in Japan found that the prevalence of NAFLD declined progressively with increasing sleep duration, with the lowest prevalence in the subgroup with 6 to ≤7 h of sleep and the highest in the groups with ≤6 and >8 h of sleep (94). A study of 708 non-diabetic adolescents found that sleep deprivation was associated with the presence of NAFLD in the younger population (95). Patients with sleep-disordered SD have higher risk of NAFLD in a Taiwanese population-based study (96). In addition, poor sleep patterns were associated with a high risk of MAFLD and severe fibrosis, and sleep difficulties, snoring, excessive daytime sleepiness, and sleep apnea symptoms were positively associated with the odds of MAFLD when specific factors of sleep patterns were examined in isolation (97). Good sleep habits, therefore, help reduce the prevalence of MAFLD.

#### 3.2.3 Stress

The stress-eating relationship is mediated by the release of cortisol from the hypothalamic pituitary adrenal (HPA) axis. In a survey studying the relationship between occupational stress and NAFLD among Chinese police officers, it was found that the higher the stress, the higher the risk of developing NAFLD (98). Chronic stress linked to obesity, stress management important in treating NAFLD (99). Studies based on the Korean population have shown direct and indirect associations between psychological factors and NAFLD, depending on individual susceptibility (100). And in another study, it was also demonstrated that higher perceived stress was associated with increased prevalence of NAFLD (101). All of these studies have shown that the greater the psychological stress the greater the risk of developing NAFLD. Another recent Mendelian randomized analysis of studies points out that depression increases the prevalence of NAFLD (102). Suggesting the impact of the psychological illness on MAFLD, attention needs to be paid to managing stress and proper relaxation and stress relief in life.

#### 3.2.4 Gut microorganisms

Study proves gut microbes linked to NAFLD development (103). Study finds that gut microbial diversity declines in NAFLD, and modulating gut microbiota health may help overcome NAFLD (104). In addition, probiotic yogurt significantly improves metabolic disorders through modulating intestinal microflora and lipid metabolism and effectively regulating the occurrence and development of MAFLD (105). By improving the flora of the gut microbiota, it helps reduce the risk of developing MAFLD.

### 3.3 Environment

It has been suggested that long-term exposure to PM_2.5_ significantly increases the risk of metabolic disorders, which can increase lipid accumulation and loss of liver function (106). Ambient PM_2.5_ stems from a complex interaction of multiple emissions and chemical reactions; it is a mixture of various chemical components such as elemental carbon, organic carbon, sulfate (SO_4_^2−^), nitrate (NO_3_^−^), and ammonium (NH_4_^+^), and chronic exposure to PM_2.5_ and its five major chemical components may increase MAFLD risk. Of these, nitrate may have the greatest impact on MAFLD (107). In addition, in a study analyzing the correlation between cirrhosis and air pollution in patients with NAFLD, based on the UK population, it was noted that long-term exposure to air pollution was associated with the risk of NAFLD and cirrhosis in the UK population (108). There was a dose-dependent relationship between different fibrosis stages and PM2.5 levels (PM_2.5_ levels in patients with fibrosis stages 0, 1–2, and 3–4:27.9, 28.4, and 29.3 μg/m3, respectively; trend *p* < 0.001). Exposure to PM2.5 is associated with advanced liver fibrosis in patients with MAFLD (109). Recently, a cross-sectional study based on Taiwanese and Hong Kong populations analyzed airborne concentrations of nitrogen dioxide (NO_2_) and ozone (O_3_) and fine particulate matter (PM_2.5_) in relation to advanced fibrosis in NAFLD. The results showed that higher ambient PM_2.5_ and NO_2_ were associated with higher odds of NAFLD and advanced fibrosis, and that, in addition, lowering PM_2.5_ and NO_2_ concentrations may be an effective method of preventing NAFLD, and that further research is necessary on O_3_ (110). From this, we concluded that reducing air pollution or actively reducing exposure to PM_2.5_ can effectively prevent MAFLD.

## 4 Conclusion and outlook

MAFLD is a liver disease with high prevalence, and currently used to diagnose MAFLD is still mainly diagnosed by imaging. This review summarizes the commonly used imaging markers (MRI-PDFF, CAP) and new evaluation markers based on the original markers (FIB-4, APRI, FLI, HSI, MAFLD-S, TyG-WC, TyG-BMI, CBST). The more studied serological markers (CHI3L1, LECT2, CTSD) are also summarized, and some serological markers in combination with imaging indices help to improve the accuracy. Combined serologic and imaging diagnosis may become an effective method for clinical diagnosis of MAFLD.

However, nowadays, indicators are generally devoted to reflecting the degree of hepatic steatosis, the degree of fibrosis, or predicting MAFLD in other metabolic disorders, and there is a lack of research on specific indicators for MAFLD, which might be useful to look for specific diagnostic indicators at the molecular level.

This review summarizes the effective methods currently used for the prevention and treatment of MAFLD in terms of diet, lifestyle habits and living environment. Dietary supplements are well suited to help in the clinic, as daily diet and lifestyle habits require a great deal of adherence from the patients themselves, and the living environment is difficult to change. Current research, based on cell and animal experiments, suggests that supplements are useful in reducing liver fat accumulation, anti-inflammatory, and antioxidant.

Current treatments targeting obesity, insulin resistance, and cardiovascular aspects are effective but lack specificity for MAFLD. This may be due to the fact that the pathomechanism of MAFLD is very complex and requires further research. It is therefore important to understand the pathogenesis of MAFLD, which may help to find drugs in the future that can both improve metabolic disorders and reduce hepatic inflammation and fibrosis, as well as provide new guidelines for finding early specific diagnostic indicators.

## Author contributions

TC: Writing – original draft. XQ: Writing – original draft. JJ: Writing – review & editing. BH: Writing – review & editing.
